# Supplementary material for: Biphasic Porous Bijel-Like Structures with Hydrogel Domains as Controlled Drug Delivery Systems
Source: Gels. 2024 Jan 18;10(1):72. doi: 10.3390/gels10010072 (PMC10815427; doi:10.3390/gels10010072)
Supplement: Supplementary file 1 [file gels-10-00072-s001.zip › gels-2795552-supplementary.pdf]

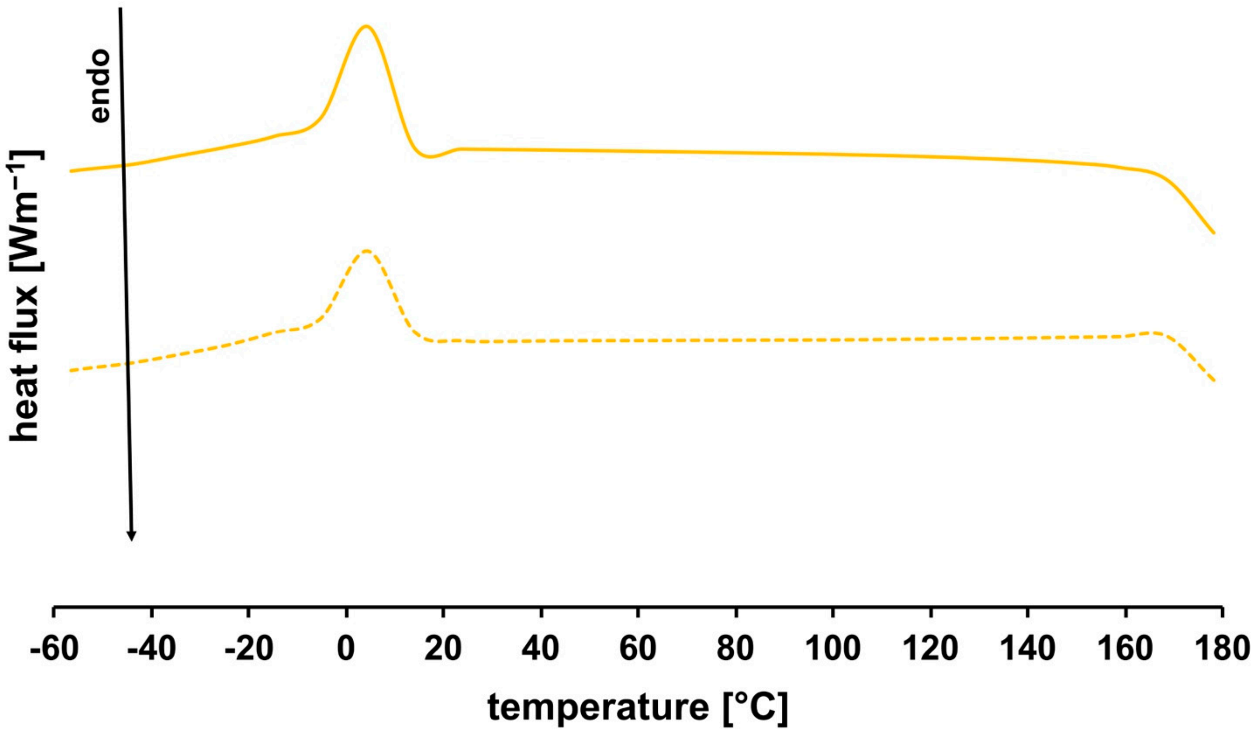

Figure S1. DSC thermogram of bijel-like samples: 10 mg/mL (yellow line), 20 mg/mL (yellow dotted line).

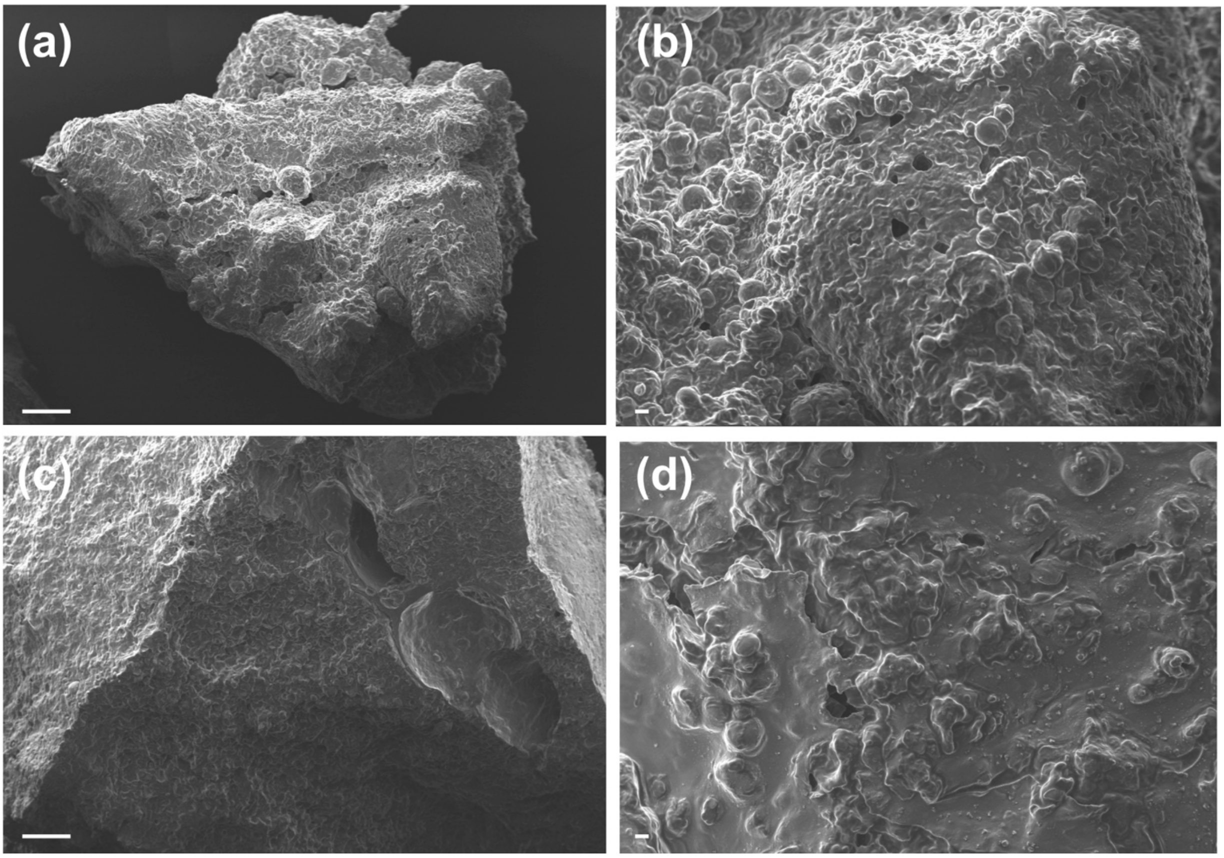

Figure S2. SEM images of different bijel samples. **a**, **b**) 20 mg/mL alginate not soaked in $CaCl_2$ (100 μm, **a**) (20 μm, **b**). **c**, **d**) 20 mg/mL alginate 3h soaked in $CaCl_2$ (100 μm, **c**) (20 μm, **d**).

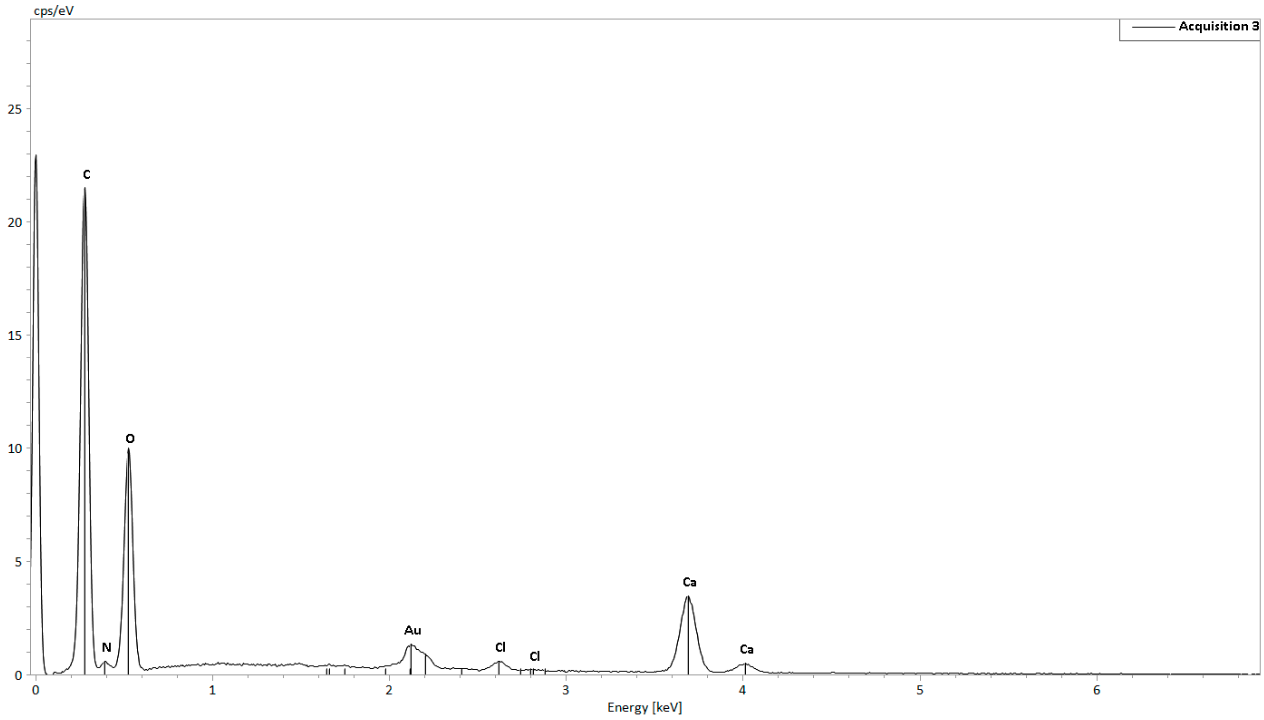

Figure S3. EDS analysis on bijel samples after soaking in $CaCl_2$ solution. The peaks related to the Ca and Cl are clearly visible, ensuring that the lumps observable through SEM analysis were $CaCl_2$ deposits.

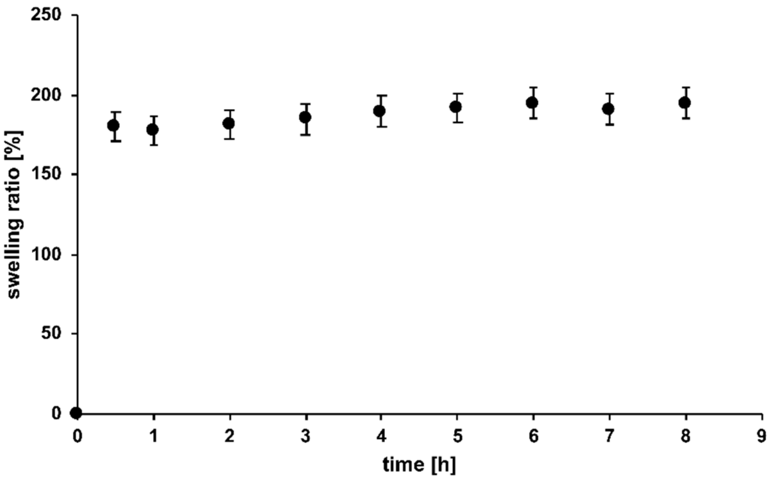

Figure S4. Swelling profile for dried bijel-like samples 20 mg/mL alginate.

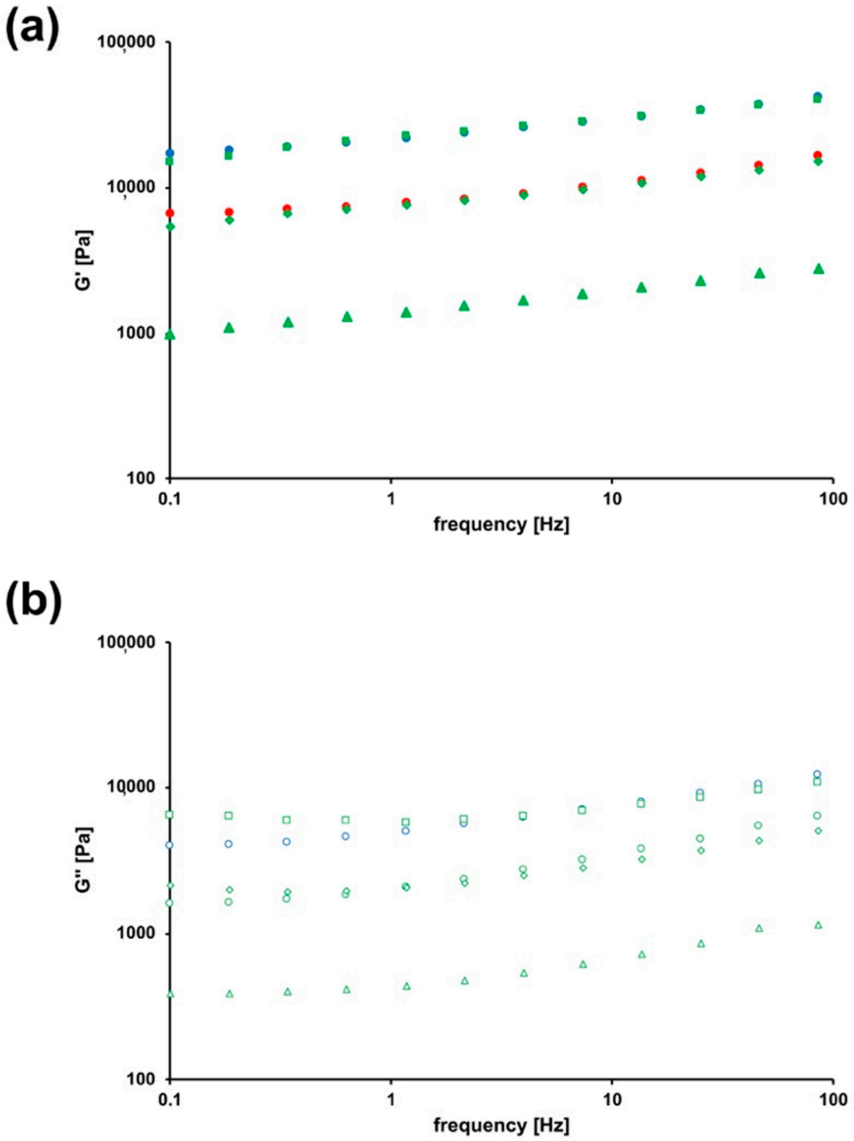

Figure S5. Frequency sweep tests for the 20 mg/mL samples: blank (blue circle), not soaked (red circle), 30 min soaking (green rhombuses), 3 h soaking (green squares) and 24 h soaking (green triangle).

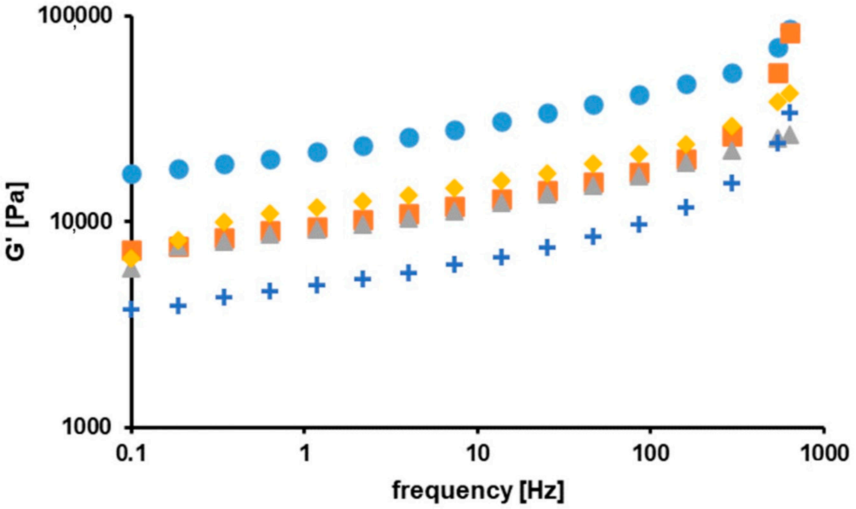

Figure S6. Frequency sweep tests for the 15 mg/mL sample. ○ blank, □ not soaked, Δ 30 min soaking, ◊ 3 h soaking and + 24 h soaking respectively.

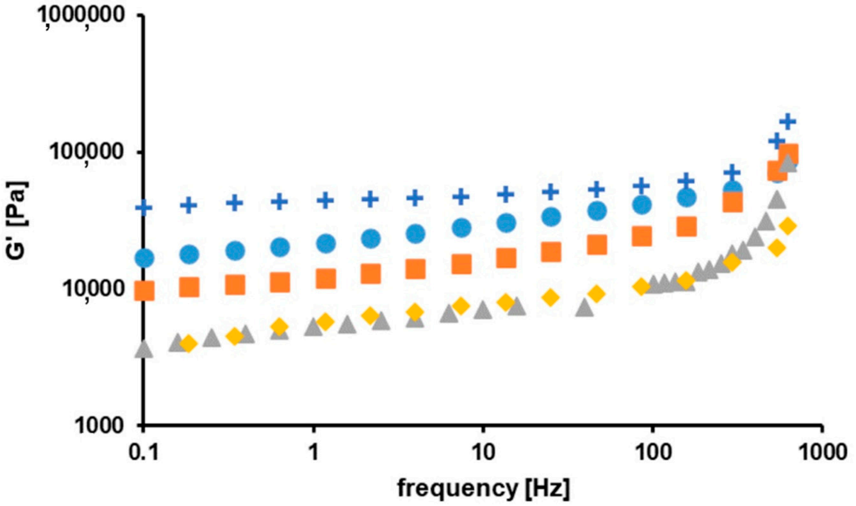

Figure S7. Frequency sweep tests for the 30 mg/mL sample. ○ blank, □ not soaked, Δ 30 min soaking, ◊ 3 h soaking and + 24 h soaking respectively.

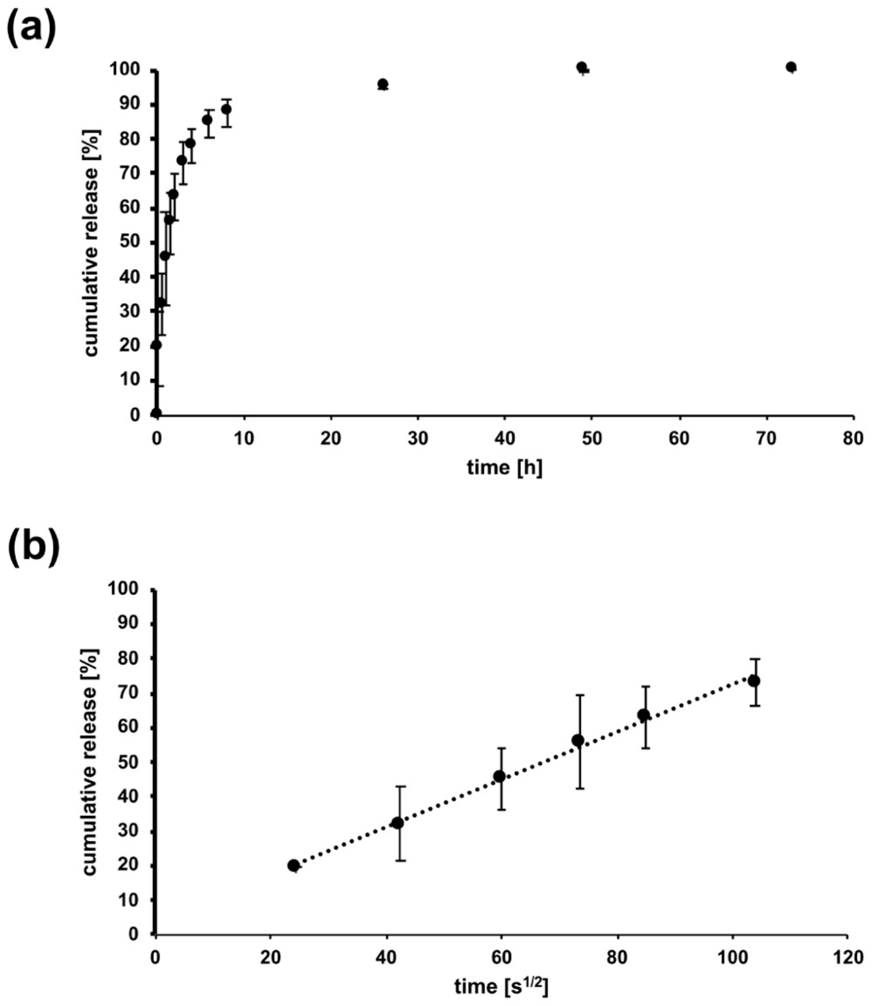

Figure S8. (**a**) Fluorescein release profile. (**b**) The slope of drug release against the square root of time is representative of Fickian diffusion coefficients for each sample ($p < 0.001$ between all groups): 20 mg/mL (black circle).

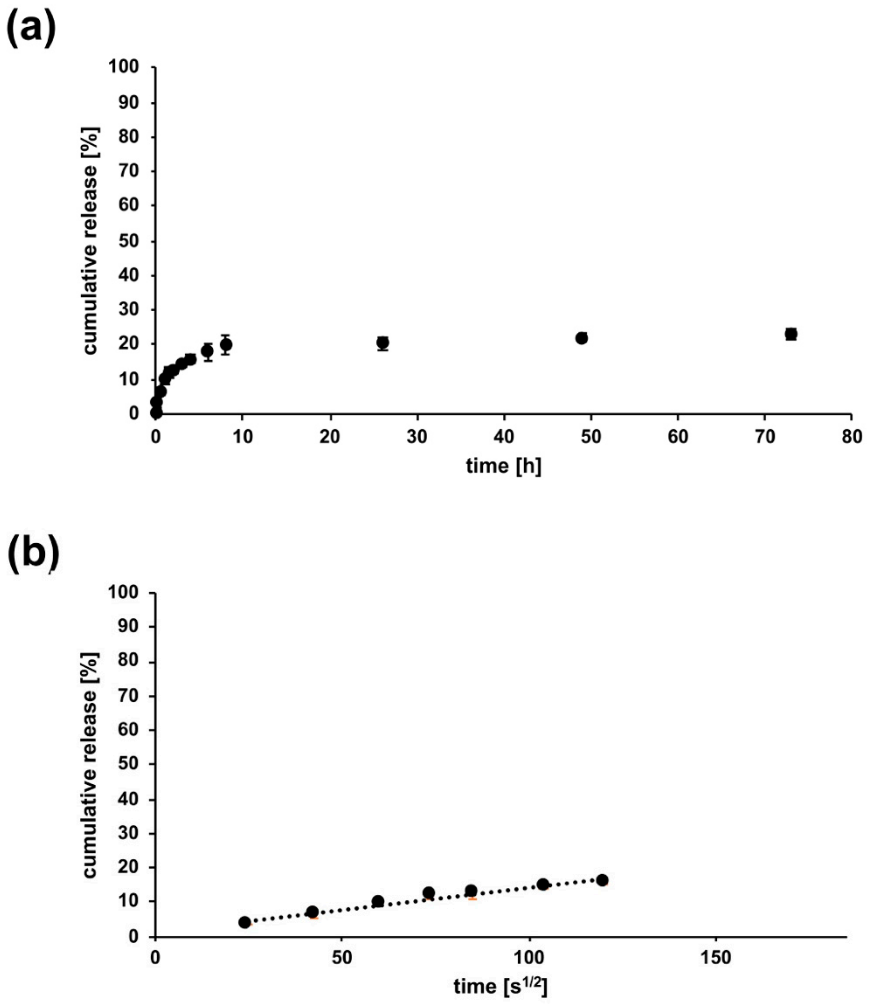

Figure S9. (**a**) Rhodamine release profile. (**b**) The slope of drug release against the square root of time is representative of Fickian diffusion coefficients for each sample ($p < 0.001$ between all groups): 20 mg/mL (black circle).

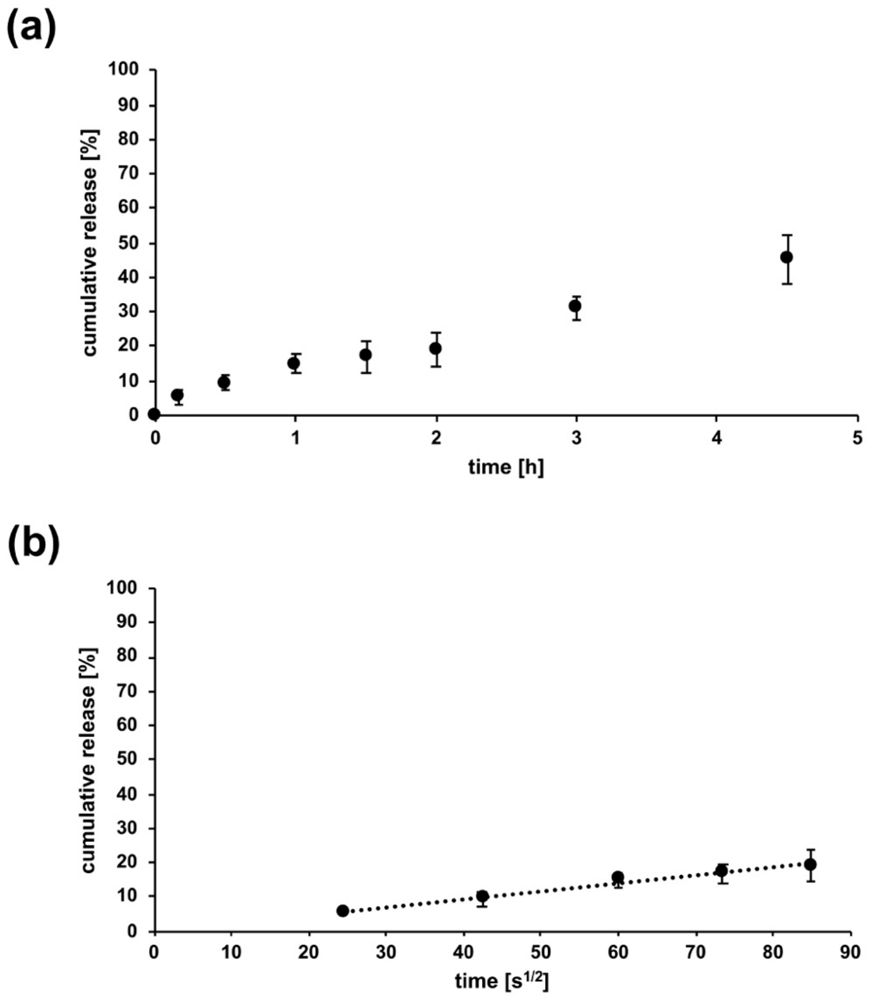

Figure S10. (**a**) FITC release profile. (**b**) The slope of drug release against the square root of time is representative of Fickian diffusion coefficients for each sample ($p < 0.001$ between all groups): 20 mg/mL (black circle).
